# Evaluation of Molecular Responses and Longevity Markers in *Acheta domesticus* Following Combined Resveratrol and Nanodiamond Exposure

**DOI:** 10.3390/ijms27062786

**Published:** 2026-03-19

**Authors:** Patrycja Ziętara-Krzyk, Barbara Flasz, Maria Augustyniak

**Affiliations:** Institute of Biology, Biotechnology and Environmental Protection, University of Silesia in Katowice, Bankowa 9, 40-007 Katowice, Poland; patrycja.zietara@us.edu.pl (P.Z.-K.); barbara.flasz@us.edu.pl (B.F.)

**Keywords:** *Acheta domesticus*, resveratrol, nanodiamonds, sirtuins, longevity

## Abstract

Sirtuins are conserved proteins regulating oxidative stress and lifespan. While they enhance cellular adaptability, the long-term biological consequences of combining bioactive compounds with nanomaterials remain poorly understood. This study examined the effects of combined resveratrol and nanodiamonds (RV+NDs) in two *Acheta domesticus* strains: wild-type (H) and longevity-selected (D). The impact was assessed across developmental stages, focusing on survival, total sirtuin activity, specific isoforms (SIRT1, SIRT6), oxidative stress, antioxidant enzymes, and DNA damage markers. RV+NDs exposure did not result in consistent lifespan extension or sustained oxidative stress. Molecular responses were strongly dictated by genetic background and age, as reflected by significant survival differences between strains H and D (*p* < 0.001) Notably, a persistent increase in total sirtuin activity (~60% ↑ across developmental stages) occurred exclusively in the longevity-selected strain, though no stable activation of SIRT1 or SIRT6 was detected. While classical redox parameters showed only transient changes, DNA damage response markers emerged as the most sensitive indicators of RV+NDs exposure. Overall, the findings demonstrate that RV+NDs treatment induces context-dependent, adaptive molecular responses. This highlights the critical role of genetic background and age in shaping ageing-related pathways, suggesting that nanodelivery systems do not produce universal effects across different genotypes.

## 1. Introduction

The ageing process is closely linked to cellular defence mechanisms and oxidative stress [1]. It is an inherently interdisciplinary phenomenon that requires combining knowledge from molecular biology, physiology, biochemistry, and toxicology [2,3]. Its complexity results from the involvement of many different cellular factors that together determine the pace of ageing and the lifespan of an organism [1].

Attention from researchers has been drawn to sirtuins—enzymes often referred to as “longevity enzymes” [4]. They form a family of NAD^+^-dependent deacetylases and ADP-ribosyltransferases that regulate numerous cellular processes, including genome stability, metabolism, stress response, and apoptosis [5,6]. In mammals, seven sirtuin isoforms (SIRT1-SIRT7) have been identified, which differ in their cellular localisation and biological roles. SIRT1, SIRT6, and SIRT7 are primarily nuclear proteins, SIRT2 is mainly found in the cytoplasm, whereas SIRT3–SIRT5 are predominantly localised in mitochondria [7,8]. Through NAD^+^-dependent deacetylation or ADP-ribosylation, sirtuins regulate chromatin structure, DNA repair, mitochondrial metabolism, and stress responses, linking cellular energy status to ageing and longevity [8,9,10]. Studies in model organisms (mainly vertebrates: mice, rats) have shown that certain sirtuins—especially SIRT1 and SIRT6—may play a direct role in regulating lifespan [11,12,13]. Moreover, it has been demonstrated that the activity of these enzymes can be modulated by natural compounds such as resveratrol (RV), which exhibits health-promoting and anti-ageing properties [14,15]. One of the significant limitations of using RV is its low bioavailability and short retention time in the body [16]. In humans, the bioavailability of RV is considered very low, and its rapid metabolism significantly limits its potential biological effects [17,18]. Literature reports indicate that although nanodelivery systems can increase the bioavailability of bioactive compounds, their pharmacokinetic and biological consequences do not always translate into changes in lifespan and are more often reflected in the regulation of molecular processes rather than in global longevity indicators [19,20]. Nevertheless, the need for drug carrier systems that could enhance the effectiveness of this compound is emphasised [17,21]. Among nanomaterials used as drug delivery vehicles, nanodiamonds exhibit several advantageous properties, including high biocompatibility, large surface area for adsorption of bioactive compounds, chemical stability, and relatively low cytotoxicity [22,23]. Nanodiamond surface chemistry enables adsorption of polyphenols such as resveratrol via hydrogen bonding and π–π interactions, potentially influencing its stability and release [24,25]. In this context, nanodiamonds (NDs) appear to be a promising solution—biocompatible nanomaterials capable of binding and gradually releasing active substances [23,26,27]. Previous studies indicate that nanodiamonds show low toxicity and can interact with insect biological systems, including *Acheta domesticus*, without causing acute lethality [4,28]. However, the effects of administering NDs combined with resveratrol (RV+NDs) through food on insect organisms have not yet been investigated, either in terms of efficacy or preliminary safety. Insects, including *Drosophila melanogaster* and *Acheta domesticus* (the house cricket), are valuable models for studying the mechanisms of ageing and the response to pharmacological interventions due to the conservation of key pathways regulating oxidative stress and longevity-related processes [29]. Although *D. melanogaster* appears to be the most widely used insect model in ageing research, *A. domesticus* provides several complementary advantages, including larger body size facilitating biochemical analyses, clearly defined developmental stages, and genetically differentiated laboratory lines, including strains selected for extended lifespan [30,31,32]. Notably, *A. domesticus*, unlike *D. melanogaster*, undergoes incomplete metamorphosis, offering an opportunity to gain additional insights into insect ageing processes. In this context, *A. domesticus* emerges as a promising, yet still underutilised, invertebrate model. Previous studies have shown that both dietary factors and environmental conditions can significantly modulate survival and functional parameters in this species, highlighting its potential in research on the mechanisms of ageing [33,34]. In turn, reviews of nanodelivery systems for RV highlight that nanoformulations improve the solubility and potentially the bioavailability of RV, but in vivo evidence for their biological efficacy is still limited and requires further evaluation in the context of mechanisms of action [20]. While numerous nanodelivery systems for resveratrol have been investigated in mammalian and cellular models, their evaluation in insects remains very limited. Although sirtuins play an essential role in the ageing process, they are not the sole indicator of longevity-related changes. Oxidative stress, defined as an imbalance between the production of reactive oxygen species (ROS) and the organism’s ability to neutralise them, is widely recognised as one of the key drivers of cellular ageing [3,35,36]. Therefore, the level of oxidative stress may serve as a sensitive marker of the potential biological effects induced by RV+NDs, providing a starting point for toxicological risk assessment.

In this study, two strains of *A. domesticus* were used: a wild-type strain (H) and a longevity-selected strain (D). The latter constitutes a valuable model for studying ageing-related mechanisms, as selection for extended lifespan results in distinct physiological and metabolic traits associated with longevity. This approach allowed comparison of responses between organisms with different lifespan within the same species. In our previous research, we demonstrated that NDs and RV individually affect survival, sirtuin activity, oxidative stress parameters, and DNA damage markers in *A. domesticus* under corresponding experimental conditions. These findings provided the biological rationale for investigating whether their combined administration would result in additive, synergistic, or qualitatively distinct molecular responses [4,37]. Based on the above, the main research question is as follows: how does the combination of RV+NDs affect sirtuin activity in *Acheta domesticus*? The primary aim of this study was to determine the impact of this combination on overall sirtuin activity, with a particular focus on specific classes, such as SIRT1 and SIRT6, and to assess potential changes in oxidative stress levels and genome stability using complementary molecular markers, including antioxidant enzymes (SOD and CAT), lipid peroxidation, and DNA damage response indicators (pATM and γH2A.X). Accordingly, the following hypotheses were tested:

**H_1_.**  *The application of RV+NDs positively influences molecular processes in Acheta domesticus, leading to increased sirtuin activity, particularly SIRT1 and/or SIRT6. NDs may modulate the bioavailability and duration of RV action, thereby enhancing its effect and potentially leading to significant changes in sirtuin activity, especially in the longevity-selected insect strain.*

**H_2_.**  *Exposure of Acheta domesticus to RV+NDs leads to an increase in oxidative stress at the cellular level, regardless of observed changes in sirtuin activity (including SIRT1 and/or SIRT6). This effect may manifest as a disruption of antioxidant balance, indicating potential toxicity of this combination even in the presence of activated SIRT-related pathways.*

## 2. Results

### 2.1. This Survival Analysis in Acheta domesticus

**Figure 1:**
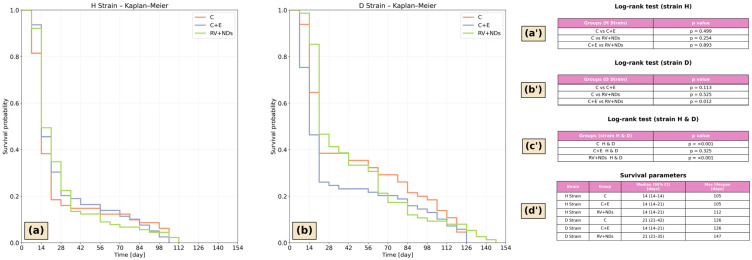
Kaplan–Meier survival analysis: (**a**) survival curves for the wild-type strain (H) monitored for up to 154 days; (**b**) survival curves for the long-lived strain (D) monitored for up to 154 days. The analysed groups included control (C), control with ethanol (C+E), and the combined resveratrol and nanodiamond treatment (RV+NDs). (**a′**,**b′**) Log-rank test results comparing survival distributions among experimental groups within each strain. (**c′**) Comparison of corresponding experimental groups between strains H and D (Python, lifelines package). (**d′**) Quantitative survival parameters derived from the Kaplan–Meier estimator implemented in Python (lifelines package), including median survival time (95% confidence intervals based on Greenwood’s variance estimate) and maximum observed lifespan for each experimental group.

In strain H, the survival curves of the individual experimental groups were largely overlapping, indicating highly similar survival patterns (Figure 1a). Overall survival in strain H was shorter compared with strain D. In contrast, strain D exhibited more pronounced divergence among survival curves across experimental groups (Figure 1b). A particularly critical period was observed between days 20 and 60, during which apparent differences in the rate of survival decline were evident among groups. During the late phase of the observation period, an extension of survival was noted, most prominently in the RV+NDs group (Figure 1b). Quantitative survival parameters derived from the Kaplan–Meier estimator indicated that in strain H, median survival was 14 days across all groups, with maximum lifespan ranging from 105 to 112 days. In strain D, median survival was 21 days in C (control) and RV+NDs (resveratrol with nanodiamonds), 14 days in C+E (control with ethanol), while the maximum lifespan reached 147 days in RV+NDs compared with 126 days in the control groups (Figure 1d′). The log-rank test identified a statistically significant difference in survival exclusively between the C+E and RV+NDs groups, and only within strain D (Figure 1b′).

A direct comparison between strains H and D revealed highly significant differences (*p* < 0.001). In both the control group and the RV+NDs group, strain D exhibited significantly longer survival compared with strain H (Figure 1a,b).

### 2.2. Superoxide Dismutase (SOD)

**Figure 2:**
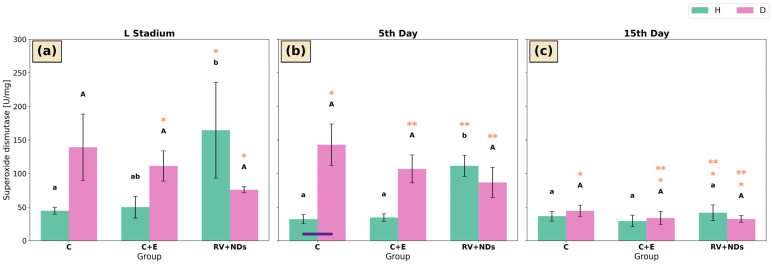
Bar charts presenting mean ± standard deviation of SOD activity (U/mg) in two *Acheta domesticus* strains (H and D) across three experimental groups: control (C), control with ethanol (C+E), and combined resveratrol and nanodiamond treatment (RV+NDs). Measurements were performed at three developmental time points: larval stage (**a**), 5th day of adulthood (**b**), and 15th day of adulthood (**c**). Statistical analysis was conducted using PERMANOVA (*p* < 0.05, corrected) and PERMDISP (*p* ≥ 0.05) in Python (scikit-bio). Lowercase letters denote significant differences among experimental groups within the same developmental stage for strain H, whereas uppercase letters indicate corresponding differences for strain D. Groups sharing at least one common letter do not differ significantly (*p* ≥ 0.05), whereas groups marked with different letters differ significantly (*p* < 0.05). Horizontal bars indicate significant differences between strains H and D within the same experimental group (*p* < 0.05). Asterisks indicate significant pairwise differences between developmental time points within the same experimental group; identical asterisks (e.g., (*)) mark the two time points that differ significantly from each other.

In the larval stage of strain H, superoxide dismutase (SOD) activity in the RV+NDs group was significantly higher than in the control (C) group, but it did not differ significantly from the C+E group (Figure 2a). A similar pattern was observed on day 5 of adulthood, when SOD activity in the RV+NDs group was significantly higher than in both the C and C+E groups (Figure 2b). However, this trend of elevated SOD activity in the RV+NDs group was not maintained over time, as it was not reproduced in measurements performed on day 15 of adulthood (Figure 2c). Concurrently, within the RV+NDs group, a significant temporal decline in SOD activity was observed, with SOD activity on day 15 of adult life being significantly lower than during the larval stage and on day 5 of adulthood (Figure 2a–c).

In insects of strain D, significant differences in SOD activity were observed exclusively between time points, indicating that RV+NDs treatment did not affect SOD activity within a given age group of strain D. In the control group, SOD activity measured on day 5 was higher than that recorded on day 15. Similarly, in both the C+E and RV+NDs groups, SOD activity at the larval stage and on day 5 was higher than that observed on day 15 (Figure 2a,c).

Moreover, only on day 5 of adult life and exclusively in the control group, was SOD activity significantly higher in strain H than in strain D (Figure 2b).

### 2.3. Percent Inhibition of SOD Reaction in Acheta domesticus

**Figure 3:**
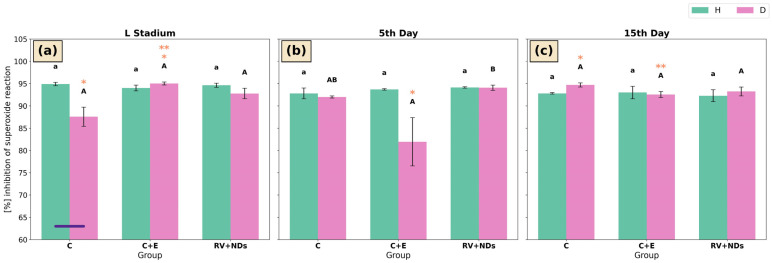
Bar charts presenting mean ± SD % inhibition of SOD reaction in *Acheta domesticus* strains H and D across experimental treatments and measurement points: (**a**) larval stage, (**b**) 5th day of adulthood, and (**c**) 15th day of adulthood. Experimental groups were control (C), control with ethanol (C+E), and combined resveratrol and nanodiamond treatment (RV+NDs). Lowercase and uppercase letters denote significant differences for strains H and D, respectively; identical asterisks mark significantly different developmental time points within the same experimental group, as defined in Figure 2.

Across the analysed time points (larval stage, day 5, and day 15 of adult life), insects of strain H exhibited comparable levels of superoxide reaction inhibition across all experimental groups (C, C+E, and RV+NDs), with no statistically significant differences attributable to the applied treatments (Figure 3a–c).

In strain D, significant intergroup differences were observed exclusively on day 5 of adult life. Specifically, the percentage of superoxide inhibition in the C+E group was significantly lower than in the RV+NDs group, whereas no difference was observed compared with the control group (Figure 3b). Temporal analysis revealed a significant increase in % inhibition in the control group of long-lived insects between the larval stage and day 15 of adulthood (Figure 3a,c). A distinct temporal pattern was observed in the C+E group, in which the highest level of superoxide reaction inhibition was recorded at the larval stage, a lower level at day 15, and the lowest level at day 5 of adult life (Figure 3a–c).

A significant difference between strains was detected only in the control group at the larval stage, where the percentage of superoxide reaction inhibition was significantly higher in strain H compared with strain D (Figure 3a).

### 2.4. Catalase (CAT)

**Figure 4:**
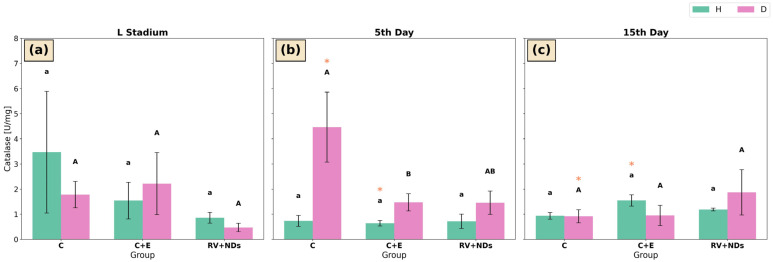
Bar charts presenting mean ± SD of catalase (CAT) activity in *Acheta domesticus* strains H and D across experimental treatments and measurement points: (**a**) larval stage, (**b**) 5th day of adulthood, and (**c**) 15th day of adulthood. Experimental groups were control (C), control with ethanol (C+E), and combined resveratrol and nanodiamond treatment (RV+NDs). Lowercase and uppercase letters denote significant differences for strains H and D, respectively; identical asterisks mark significantly different developmental time points within the same experimental group, as defined in Figure 2.

In strain H, catalase (CAT) activity did not show statistically significant differences among the experimental groups (C, C+E, and RV+NDs) at any of the analysed time points (Figure 4a–c). However, significant temporal changes were observed in the C+E group, in which CAT activity on day 15 was significantly higher than on day 5 of adult life (Figure 4b,c). In strain D, a significant reduction in CAT activity was detected only on day 5 of adult life, where CAT activity in the C+E group was significantly lower than in the control (C) group (Figure 4b). In addition, within the control group (C), a significant temporal decline in CAT activity was observed, with CAT activity on day 5 being significantly higher than that recorded on day 15 of adult life (Figure 4b,c).

### 2.5. Lipid Peroxidation (LPO)

**Figure 5:**
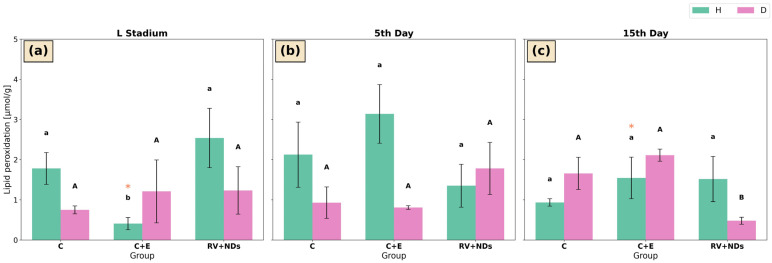
Bar charts presenting mean ± SD of lipid peroxidation (LPO) level in *Acheta domesticus* strains H and D across experimental treatments and measurement points: (**a**) larval stage, (**b**) 5th day of adulthood, and (**c**) 15th day of adulthood. Experimental groups were control (C), control with ethanol (C+E), and combined resveratrol and nanodiamond treatment (RV+NDs). Lowercase and uppercase letters denote significant differences for strains H and D, respectively; identical asterisks mark significantly different developmental time points within the same experimental group, as defined in Figure 2.

In strain H, the only significant difference in lipid peroxidation (LPO) levels was the lower LPO level observed in the C+E group at the larval stage compared with the C and RV+NDs groups (Figure 5a). No significant differences among experimental groups were detected on day 5 or day 15 of adult life (Figure 5b,c). However, within the C+E group of strain H, LPO levels on day 15 of adult life were significantly higher than those measured at the larval stage (Figure 5a,c).

In strain D, LPO levels did not differ significantly among experimental groups at the larval stage or on day 5 of adult life. Changes were observed only on day 15 of adult life, when LPO levels in the RV+NDs group were significantly lower than those in the C and C+E groups (Figure 5c).

### 2.6. Sirtuins

#### 2.6.1. Universal Sirtuin Activity

**Figure 6:**
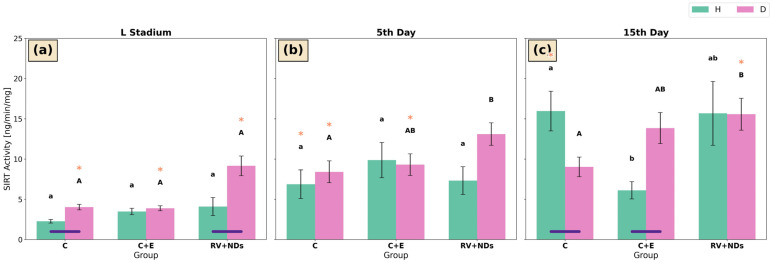
Bar charts presenting mean ± SD of sirtuin activity in *Acheta domesticus* strains H and D across experimental treatments and measurement points: (**a**) larval stage, (**b**) 5th day of adulthood, and (**c**) 15th day of adulthood. Experimental groups were control (C), control with ethanol (C+E), and combined resveratrol and nanodiamond treatment (RV+NDs). Lowercase and uppercase letters denote significant differences for strains H and D, respectively; identical asterisks mark significantly different developmental time points within the same experimental group, as defined in Figure 2.

At the larval stage and on day 5 of adult life in strain H, no significant differences in sirtuin activity were detected among the experimental groups (C, C+E, and RV+NDs) (Figure 6a,b). On day 15 of adult life, a significant reduction in sirtuin activity was observed in the C+E group compared with the control group (Figure 6c). Concurrently, in strain H, sirtuin activity in the control group was significantly higher on day 15 than on day 5 of adult life (Figure 6b,c).

In strain D, the first significant intergroup difference was observed on day 5 of adult life, when sirtuin activity in the control group was significantly lower than in the RV+NDs group (Figure 6b). This pattern persisted on day 15 of adult life (Figure 6c). Moreover, temporal analysis revealed a significant increase in sirtuin activity in the C and C+E groups between the larval stage and day 5 of adult life (Figure 6a,b), whereas in the RV+NDs group, sirtuin activity on day 15 was significantly higher than at the larval stage (Figure 6a,c).

Numerous inter-strain differences were also observed. At the larval stage, sirtuin activity in strain H was significantly lower than in strain D in both the control and RV+NDs groups (Figure 6a). On day 15 of adult life, higher sirtuin activity was recorded in strain H than in strain D in the control group; in contrast, the opposite relationship was observed in the C+E group, where sirtuin activity was significantly higher in strain D than in strain H (Figure 6c).

#### 2.6.2. Sirtuin 1 Activity

**Figure 7:**
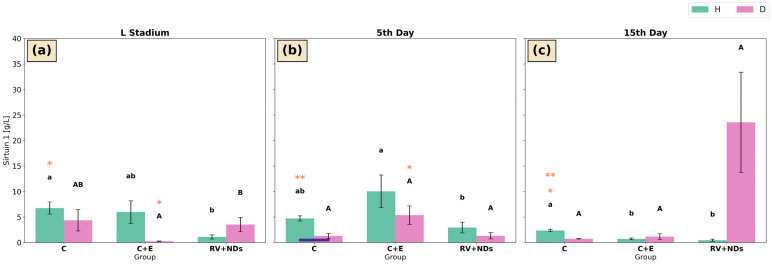
Bar charts presenting mean ± SD of Sirtuin 1 activity in *Acheta domesticus* strains H and D across experimental treatments and measurement points: (**a**) larval stage, (**b**) 5th day of adulthood, and (**c**) 15th day of adulthood. Experimental groups were control (C), control with ethanol (C+E), and combined resveratrol and nanodiamond treatment (RV+NDs). Lowercase and uppercase letters denote significant differences for strains H and D, respectively; identical asterisks mark significantly different developmental time points within the same experimental group, as defined in Figure 2.

At the larval stage in strain H, sirtuin 1 activity in the control group was significantly higher than in the RV+NDs group (Figure 7a). In contrast, on day 5 of adult life, sirtuin 1 activity in the C+E group was significantly higher than in the RV+NDs group, while showing no significant difference relative to the control group (Figure 7b). On day 15 of adult life, sirtuin 1 activity in the control group was significantly higher than in both remaining experimental groups (C+E and RV+NDs) (Figure 7c). Temporal analysis revealed a significant decrease in sirtuin 1 activity in the control group on day 15 compared with the control group at the larval stage and on day 5 of adult life (Figure 7a–c).

In strain D, the only significant differences in sirtuin 1 activity were observed at the larval stage, where activity in the RV+NDs group was significantly higher than in the C+E group (Figure 7a). In addition, a significant temporal increase in sirtuin 1 activity between the larval stage and day 5 of adult life was detected in the C+E group (Figure 7a,b).

Differences between strains H and D were observed exclusively on day 5 of adult life in the control group, where sirtuin 1 activity was significantly higher in strain H compared with strain D (Figure 7b).

#### 2.6.3. Sirtuin 6 Activity

**Figure 8:**
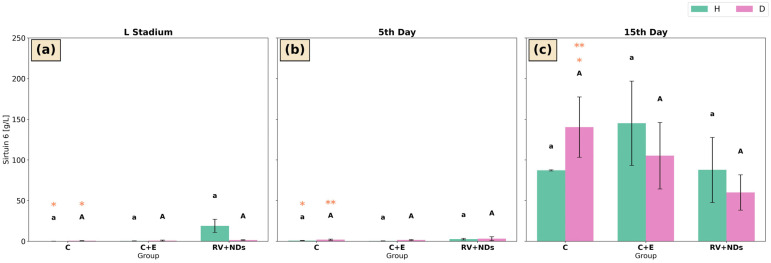
Bar charts presenting mean ± SD of Sirtuin 6 activity in *Acheta domesticus* strains H and D across experimental treatments and measurement points: (**a**) larval stage, (**b**) 5th day of adulthood, and (**c**) 15th day of adulthood. Experimental groups were control (C), control with ethanol (C+E), and combined resveratrol and nanodiamond treatment (RV+NDs). Lowercase and uppercase letters denote significant differences for strains H and D, respectively; identical asterisks mark significantly different developmental time points within the same experimental group, as defined in Figure 2.

In both strain H and strain D, no statistically significant differences were observed among the experimental groups (C, C+E, and RV+NDs) at any of the analysed time points (Figure 8a–c). Nevertheless, in strain H, a significant temporal increase in activity was recorded in the control group between the larval stage and day 5 of adult life (Figure 8a,b). In strain D, significant temporal changes were also observed in the control group, including a significant increase in activity between the larval stage and day 15 of adult life (Figure 8a,c) and between day 5 and day 15 of adult life (Figure 8b,c).

### 2.7. DNA Damage

#### 2.7.1. Double-Strand Break (DSB)

**Figure 9:**
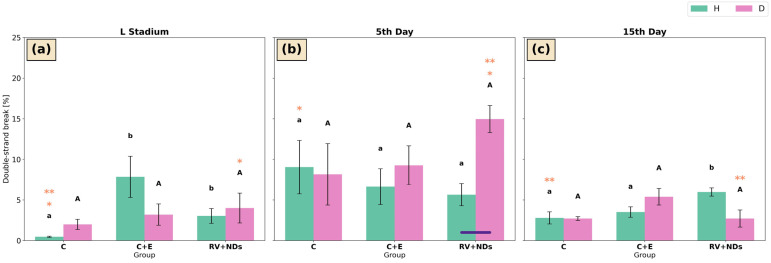
Bar charts presenting mean ± SD of double-strand break (DSB) in *Acheta domesticus* strains H and D across experimental treatments and measurement points: (**a**) larval stage, (**b**) 5th day of adulthood, and (**c**) 15th day of adulthood. Experimental groups were control (C), control with ethanol (C+E), and combined resveratrol and nanodiamond treatment (RV+NDs). Lowercase and uppercase letters denote significant differences for strains H and D, respectively; identical asterisks mark significantly different developmental time points within the same experimental group, as defined in Figure 2.

At the larval stage in strain H, the level of DNA double-strand breaks (DSBs) differed significantly among the experimental groups. The lowest percentage of DSBs was observed in the control group, whereas significantly higher values were detected in the C+E and RV+NDs groups (Figure 9a). Another significant intergroup difference in DSB levels in strain H was observed only on day 15 of adult life, when the highest level of DNA damage was detected in the RV+NDs group and the lowest in the control group (Figure 9c). In addition, temporal analysis revealed that at the larval stage, the percentage of DSBs in the control group was significantly lower than the values recorded on days 5 and 15 of adult life (Figure 9a–c).

In strain D, no significant differences among the experimental groups were detected at any of the analysed time points (Figure 9a). Nevertheless, temporal analysis demonstrated a significant increase in DSB levels on day 5 of adult life compared with the larval stage in the RV+NDs group (Figure 9a,b), followed by a significant decrease on day 15 relative to day 5 of adult life (Figure 9b,c).

Comparison between strains revealed that, on day 5 of adult life, DSB levels were significantly lower in strain H than in strain D in the RV+NDs group (Figure 9b).

#### 2.7.2. H2A.X

**Figure 10:**
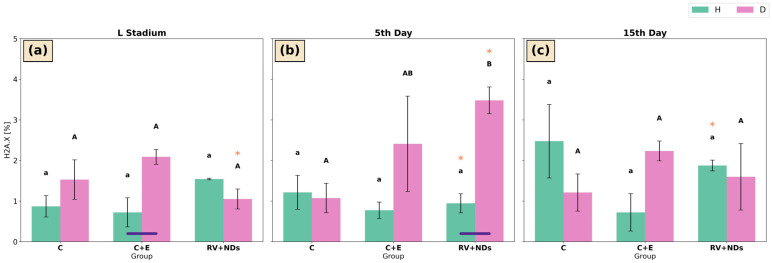
Bar charts presenting mean ± SD of H2A.X in *Acheta domesticus* strains H and D across experimental treatments and measurement points: (**a**) larval stage, (**b**) 5th day of adulthood, and (**c**) 15th day of adulthood. Experimental groups were control (C), control with ethanol (C+E), and combined resveratrol and nanodiamond treatment (RV+NDs). Lowercase and uppercase letters denote significant differences for strains H and D, respectively; identical asterisks mark significantly different developmental time points within the same experimental group, as defined in Figure 2.

In strain H, no significant differences in H2A.X levels were detected among the experimental groups (C, C+E, and RV+NDs) at any of the analysed developmental stages (Figure 10a–c). Temporal analysis indicated a significantly higher level of H2A.X phosphorylation in the RV+NDs group on day 15 compared with individuals on day 5 of adult life (Figure 10b,c).

In strain D, significant intergroup differences were observed only on day 5 of adult life, with the highest H2A.X level detected in the RV+NDs group, which was significantly higher than that in the control group (Figure 10b). A significant increase in H2A.X levels on day 5 compared with the larval stage was also observed in the RV+NDs group (Figure 10a,b).

Comparison between strains H and D revealed that at the larval stage, H2A.X levels were significantly higher in strain D than in strain H in the C+E group (Figure 10a). On day 5 of adult life, H2A.X levels in the RV+NDs group were also significantly higher in strain D compared with strain H (Figure 10b).

#### 2.7.3. pATM

**Figure 11:**
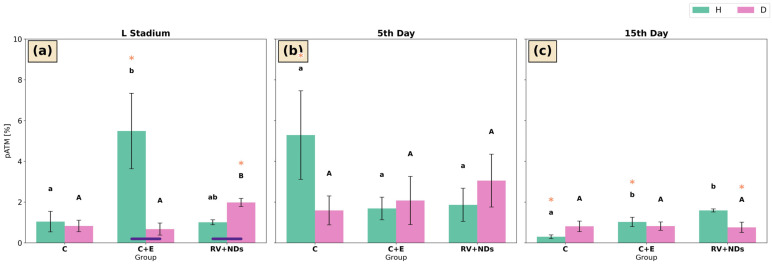
Bar charts presenting mean ± SD pATM in *Acheta domesticus* strains H and D across experimental treatments and measurement points: (**a**) larval stage, (**b**) 5th day of adulthood, and (**c**) 15th day of adulthood. Experimental groups were control (C), control with ethanol (C+E), and combined resveratrol and nanodiamond treatment (RV+NDs). Lowercase and uppercase letters denote significant differences for strains H and D, respectively; identical asterisks mark significantly different developmental time points within the same experimental group, as defined in Figure 2.

In strain H, at the larval stage the highest pATM activity was recorded in the C+E group, where it was significantly higher than in the control group (Figure 11a). Subsequently, on day 15 of adult life, pATM levels exhibited significant intergroup variation, with the highest values observed in the RV+NDs group and the lowest in the control group (Figure 11c). Temporal analysis revealed a significant decrease in pATM levels in the C+E group on day 15 compared with the larval stage (Figure 11a,c) as well as in the control group, where pATM levels were significantly lower in 15-day-old insects than in 5-day-old individuals (Figure 11b,c).

In strain D, a significant difference was observed only at the larval stage in the RV+NDs group, where pATM levels were increased relative to both the control and C+E groups (Figure 11a). Temporal analysis demonstrated a significant decrease in pATM levels on day 15 compared with the larval stage exclusively in the RV+NDs group (Figure 11a,c).

Comparison between strains H and D revealed significant differences only at the larval stage. pATM levels were significantly higher in strain H than in strain D in the C+E group (Figure 11a). In contrast, in the RV+NDs group at the same developmental stage, an opposite pattern was observed, with pATM levels being significantly lower in strain H compared with strain D (Figure 11a).

To further examine the potential relationship between sirtuin activity and DNA damage markers, Spearman’s rank correlation analysis was performed separately for strains H and D. The obtained correlation coefficients between total sirtuin activity, Sirtuin 1, or Sirtuin 6 and DNA damage markers (pATM, H2A.X, and DSB) were weak and not statistically significant (*p* ≥ 0.05) (Supplementary Figure S1).

## 3. Discussion

Previous studies on ageing in *Acheta domesticus* have focused primarily on describing age-related behavioural and functional changes, as well as on analyses of individual environmental or dietary factors. In contrast, the impact of combined interventions on molecular mechanisms associated with longevity mainly remains unexplored [33,38]. Consequently, an important step was to investigate the effects of individual bioactive factors that may influence broadly defined “longevity”.

In our earlier studies, RV and NDs were analysed exclusively as independent treatments under analogous experimental conditions, which allowed for separate assessment of their effects on sirtuin activity, oxidative stress parameters, and markers of DNA damage. The present work extends this framework by examining whether their combined administration generates a distinct regulatory profile rather than a simple additive response [4,37]. The results indicated that both compounds elicited transient responses that were strongly dependent on age and strain, without a clear or sustained effect on lifespan or stable modulation of sirtuin activity. At the same time, the occasionally divergent directions of change induced by RV and NDs suggested that their simultaneous administration might influence the pattern of the analysed parameters. On this basis, two research hypotheses were formulated: first, that combined administration of RV and NDs (RV+NDs) may modulate molecular processes associated with longevity in *Acheta domesticus*, including sirtuin activity (SIRT1 and/or SIRT6); second, exposure to RV+NDs may lead to an enhancement of oxidative stress at the cellular level, independently of changes in sirtuin activity. Given that NDs are considered potential modifiers of the bioavailability of bioactive compounds and as carriers [23,39], a subsequent stage of research was therefore designed to evaluate the effects of simultaneous RV and NDs administration on the analysed parameters.

### 3.1. Influence on Molecular Processes in Acheta domesticus

Verification of the hypotheses began with an analysis of survival, treated as an indicator of the long-term effects of the studied factors (Figure 1). The results showed that combined administration of RV and NDs did not significantly affect the lifespan of individuals from strain H (Figure 1a), whereas in strain D (Figure 1b), significant differences in the survival curves were observed. This outcome suggests that the effects of RV+NDs may differ across genetic backgrounds and do not necessarily reflect a simple additive effect of the two factors. It should also be noted that ethanol was used as a solvent for resveratrol preparation and was therefore included in the C+E group as an additional control. Although all feed variants were thoroughly dried prior to administration, which may have resulted in ethanol evaporation, the C+E group was intentionally retained to control for potential solvent-related effects. The inclusion of a vehicle control helps to distinguish the biological effects of the tested compound from potential responses associated with the solvent used for its preparation [40,41]. Importantly, no consistent differences between the C and C+E groups were observed across the analysed parameters, suggesting that the contribution of ethanol to the observed responses was likely limited. In this context, it is first worth referring to reports on resveratrol administered alone, which have demonstrated that its impact on lifespan is strongly dependent on genotype, environmental conditions, and age, and that the absence of an apparent pro-longevity effect is more the rule than the exception. For example, studies by Staats et al. showed that dietary RV did not significantly affect survival, oxidative stress, or the expression of longevity-related genes under standard conditions in *D. melanogaster* [42]. In contrast, Wang et al. demonstrated that the effect of RV on *D. melanogaster* lifespan was observed only under strictly defined dietary conditions, particularly those with altered nutrient composition, and that the response depended on sex and the protein-to-carbohydrate ratio. By contrast, no such effect was observed under standard, dietary restriction, or high-sugar low-protein diets [43].

Similarly, studies on nanodelivery systems have shown that improved bioavailability of bioactive compounds does not necessarily translate directly into changes in survival but may instead manifest at the level of molecular process regulation [44,45]. Although various types of nanomaterials, including polymeric, metallic, and lipid-based carriers, enhance the stability, solubility, and absorption of RV, their actual biological effectiveness remains dependent on the type of carrier and the application context and does not always result in unequivocal therapeutic effects in in vivo studies [17,46]. Moreover, pharmacokinetic studies have demonstrated that encapsulation of RV in nanoparticles significantly increases its pharmacokinetic bioavailability (AUC, t_1_/_2_), without assuming a direct translation of this effect into improved survival [47]. Considering these findings, further analyses focused on molecular responses, including sirtuin activity, oxidative stress markers, and indicators of DNA damage. Recent studies also highlight that nanodiamonds exhibit favourable biological properties, including high biocompatibility and a large surface area enabling interaction with bioactive molecules, which supports their increasing exploration in various biomedical applications, particularly in drug delivery systems [48,49,50].

This analysis showed that simultaneous administration of RV+NDs resulted in a qualitatively different molecular response compared with that observed when each factor was applied separately. In contrast to previous studies in which RV and NDs induced numerous, often inconsistent changes in oxidative stress parameters and DNA damage markers [37], the RV+NDs combination did not cause a sustained increase in lipid peroxidation (Figure 5) or significant deregulation of SOD and CAT activity (Figure 2 and Figure 4). The observed changes were transient and strongly dependent on age and strain. This is consistent with reports indicating that the in vivo action of RV may not involve a persistent reduction in reactive oxygen species levels but rather modulation of signalling pathways and stress responses in a manner dependent on dose, organismal age, and metabolic state [43,51]. From this perspective, the presence of NDs in the RV+NDs system may stabilise RV and modify its interaction kinetics, thereby limiting the variability of oxidative responses observed when the compound is administered in its free form [22,45,52]. Beyond acting as a carrier, NDs may exert a hormetic effect, where their presence induces a mild mechanical or chemical stress that may prime the antioxidant defence system of *A. domesticus*. This potentially explains the lack of sustained lipid peroxidation in the RV+NDs group (Figure 5). Furthermore, the large surface area of NDs, characterised by various functional groups, may actively scavenge free radicals or interact with cellular proteins, further modulating the redox state and limiting the variability of oxidative responses [53,54].

**Figure 12:**
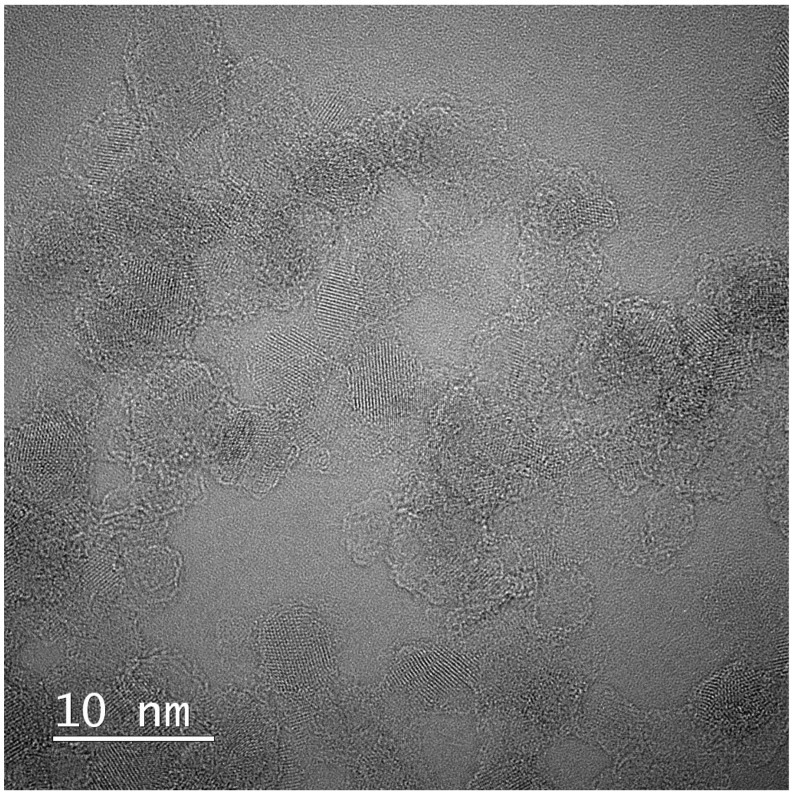
HR-TEM micrograph of nanodiamonds (NDs; Single-Digit Nanodiamonds, 50 mg mL^−1^, PlasmaChem GmbH, Germany) showing lattice fringes characteristic of crystalline nanodiamond structures (TITAN Cubed, Thermo Fisher Scientific, USA).

**Figure 13:**
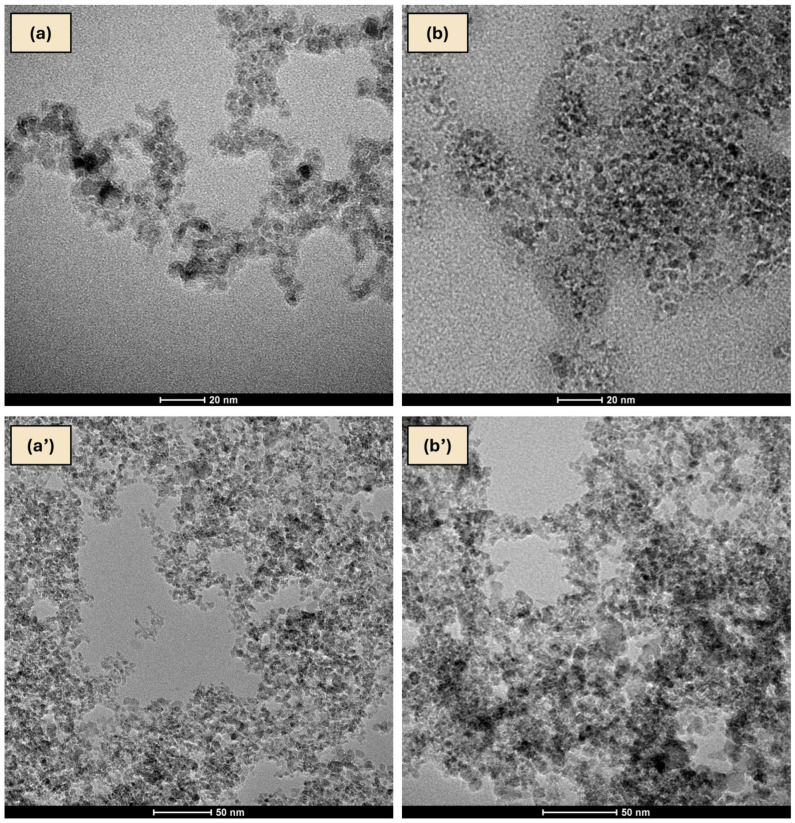
TEM micrographs of nanodiamonds (NDs; Single-Digit Nanodiamonds, 50 mg mL^−1^, PlasmaChem GmbH, Germany) at scales (**a**) 20 nm and (**a**′) 50 nm, as well as RV+NDs (resveratrol with nanodiamonds) at scales (**b**) 20 nm and (**b**′) 50 nm (TECNAI G2 X-TWIN, Thermo Fisher Scientific, USA).

**Figure 14:**
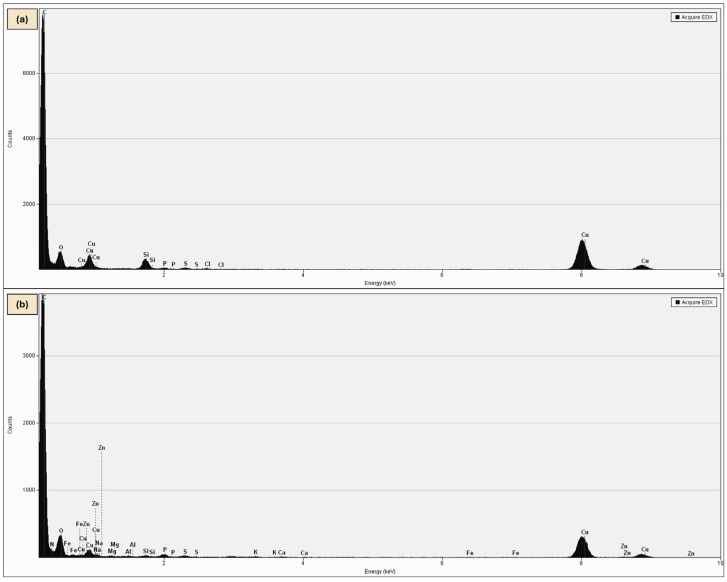
EDS spectrum of (**a**) NDs; (**b**) RV+NDs (SEM-FIB Helios NanoLab 450HP, Thermo Fisher Scientific, USA). Abbreviations provided in Figure 13.

HR-TEM image (Figure 12) revealed well-defined lattice fringes forming a regular crystalline pattern typical for nanodiamond nanocrystallites. This interpretation is supported by TEM and EDS analyses, which demonstrated changes in the degree of aggregation and surface characteristics of NDs in the presence of RV (Figure 13 and Figure 14). Similar ordered arrangements corresponding to the cubic crystal structure of diamond and parallel crystallographic planes have been widely described in HR-TEM studies of NDs [55,56]. UV–Vis spectra confirmed the characteristic absorption of RV and enabled estimation of the fraction of non-bound RV in the supernatant, allowing determination of the loading efficiency of RV on NDs (Supplementary Figures S2 and S3). Moreover, DLS and zeta potential analyses together provided additional evidence suggesting interactions between RV molecules and the NDs surface (Supplementary Figures S4 and S5). The Raman spectrum of NDs showed broad bands in the region of approximately 1350 and 1580–1600 cm^−1^, which is characteristic of detonation nanodiamonds and is commonly attributed to D and G bands of surface-related sp^2^ carbon surrounding the diamond core [57,58,59,60]. The absence of a sharp bulk-diamond peak at 1332 cm^−1^ should not be considered unexpected, since in very small NDs, particularly sub-10 nm particles, this band is often broadened, shifted, or poorly resolved due to phonon confinement and the complex core–shell structure of detonation NDs [57,58]. In the RV+NDs, attenuation of ND-related bands together with the appearance of a shifted feature in the high-wavenumber region may suggest modification of the nanodiamond surface environment following RV adsorption. Although Raman spectroscopy alone does not provide definitive proof of a stable complex, these changes, together with UV–Vis loading analysis and the reduction in zeta potential, are consistent with the formation of an RV associated with NDs based on surface interaction. The reduced Raman intensity may reflect adsorption-induced changes in Raman scattering efficiency related to modifications in molecular polarizability and the local electronic environment at the nanodiamond surface [61,62]. At the same time, it should be emphasised that for NDs, the size and degree of aggregation observed by electron microscopy do not necessarily directly correspond to particle behaviour in solution. The DLS method determines the hydrodynamic diameter, which includes the hydration layer and loosely associated particle clusters, often resulting in values substantially larger than the sizes observed by TEM [28,63,64]. These physicochemical analyses provide an initial characterisation of the RV+NDs and confirm the interaction between both components, including changes in aggregation behaviour and surface properties. More detailed characterisation of parameters such as encapsulation efficiency and release kinetics would require additional targeted experiments and therefore remains a direction for future investigation.

Additionally, a clear activation of DNA damage response markers (pATM, γH2A.X, and DSBs) was observed (Figure 9, Figure 10 and Figure 11). The concurrent activation of DNA damage response markers such as pATM and γH2A.X suggests that this response may initiate DDR (DNA damage response) mechanisms, which may secondarily modulate total sirtuin activity without selectively activating individual isoforms [65,66,67]. Dobbin et al. demonstrated that ATM activation in response to DNA damage leads to secondary regulation of SIRT1 activity [68]. Furthermore, Tang et al. described a mechanism in which SIRT7 regulates ATM activity during the attenuation phase of the DNA damage response, indicating functional coupling between DDR pathways and sirtuin activity [69]. In turn, according to Song et al., SIRT6 may be activated in response to DNA damage, with its enzymatic activity depending on the nature and dynamics of the damage [70]. Rizzo et al. further showed that SIRT6 is involved in regulating the DNA damage response by influencing the stability of components that maintain genome integrity, particularly under conditions of replication stress [71]. These findings indicate that SIRT6’s role in DDR is regulatory and context-dependent rather than sustained activation. Although no statistically significant correlations were detected, the concurrent activation of DNA damage response markers and increased total sirtuin activity in strain D suggests a potential indirect relationship between these processes. However, this observation requires further investigation to clarify the underlying mechanisms. Importantly, in contrast to the effects observed following administration of RV or NDs alone, these changes did not show a tendency to accumulate with age [37], suggesting that the response induced by RV+NDs may reflect adaptive mechanisms rather than progressive damage. It should also be noted that the present analysis was limited to an early time window (up to day 15 of adult life). Therefore, potential long-term effects such as gradual ND accumulation and its possible influence on DNA repair processes cannot be excluded, especially considering that studies in mammalian models indicate that nanodiamonds may persist in tissues during prolonged exposure [72,73]. Accordingly, in the subsequent stage, an attempt was made to evaluate sirtuin activity as a potential mechanistic contributor to the observed changes.

### 3.2. Observed Changes in Sirtuin Activity

The analysis of total sirtuin activity (Figure 6) revealed differential responses to combined RV+NDs administration in the examined *A. domesticus* strains (H and D). The absence of a pronounced response in strain H and the sustained sirtuin activation in strain D indicate that the effect of RV+NDs may depend on genetic background and manifest only under specific conditions. This suggests that selection for longevity is associated with altered sensitivity of regulatory systems controlling sirtuin activity and the molecular stress response, which may underlie strain D’s susceptibility to the modulatory effects of RV+NDs. Notably, the literature has highlighted a significant influence of genetic background on the observed effects of factors modulating SIRT pathway activity, which may account for their manifestation in only a specific strain [74]. Importantly, the distinct nature of this response compared with the effects of RV or NDs administered individually indicates that the simultaneous application of both substances leads to altered sirtuin regulation rather than a simple amplification of the effects of each alone [37].

The analysis of the examined sirtuin isoforms showed that the observed increase in total sirtuin activity was not associated with unequivocal activation of either SIRT1 or SIRT6 (Figure 7 and Figure 8). The activities of both sirtuins showed only limited, stage-specific changes, which, in strain D, were not sustained over time. Moreover, SIRT6 activity remained essentially unchanged across experimental groups, and the observed differences were primarily attributable to individual age. This distribution of effects suggests that the RV+NDs combination does not selectively activate individual sirtuin isoforms but may instead modulate the activity of this enzyme family indirectly in a developmentally dependent manner. It should also be considered that total sirtuin activity reflects the combined contribution of multiple sirtuin isoforms, not exclusively SIRT1 or SIRT6. In addition to nuclear sirtuins, cytoplasmic and mitochondrial members of this family (e.g., SIRT2, SIRT3, SIRT4, and SIRT5) are involved in cellular stress responses and metabolic regulation [75,76]. In invertebrate models, mitochondrial sirtuins have been shown to participate in the regulation of oxidative metabolism and ROS detoxification, particularly under conditions of metabolic or oxidative stress [77,78]. For example, SIRT3-related pathways are associated with the regulation of mitochondrial antioxidant enzymes and metabolic adaptation, while SIRT2 has been linked to cytoskeletal dynamics and cellular stress responses [79,80]. Therefore, the observed increase in total sirtuin activity may reflect a broader activation of the sirtuin family rather than selective modulation of SIRT1 or SIRT6 alone.

In contrast to previous studies [37], the present results indicate that simultaneous administration of RV+NDs is associated with a distinct regulatory profile of SIRT1 and SIRT6. The observed effect may result from the cumulative regulation of multiple SIRTs or from changes in the availability of the NAD^+^ cofactor, which constitutes a common determinant of the enzymatic activity of the entire sirtuin family, irrespective of selective activation of SIRT1 or SIRT6 [81].

Literature reviews on sirtuin activators emphasise that many compounds previously regarded as activators (including resveratrol) act through indirect mechanisms, often by modulating NAD^+^ and AMPK levels rather than directly activating a specific enzymatic isoform [7,82,83]. However, SIRT1 activation is not always correlated with these factors or with metabolic or stress-related effects, as demonstrated in rat models [84]. The transient changes in SIRT1 activity observed here appear to be consistent with findings from mammalian models, in which SIRT1 expression and activity have been shown to vary in an age- and physiological state-dependent manner, often declining during later stages of life [85]. In insect models, the role of the SIRT1 ortholog (dSir2) in lifespan regulation has been shown to be highly context dependent. In *D. melanogaster*, alterations in dSir2 activity are not linearly correlated with lifespan and may reflect adaptive metabolic responses rather than sustained activation of longevity pathways [86]. At the same time, in *C. elegans* and *D. melanogaster*, when genetic background and appropriate controls were considered, dSir2 overexpression did not result in persistent lifespan extension, and previously reported effects were largely attributable to confounding factors unrelated to this sirtuin’s activity [87].

Similarly, regarding SIRT6, Kanfi et al. demonstrated that SIRT6 activity and function in mice are dependent on organismal age and physiological context, and that its overexpression or activation does not always result in linear pro-longevity effects [88]. In turn, Zhao and Wu showed in a chondrocyte model that SIRT6 regulates mitochondrial number, ROS levels, and antioxidant enzymes, while SIRT6 activity itself changes with the progression of ageing [89]. In the *Drosophila melanogaster* model, it has been shown that the SIRT6 ortholog (dSirt6) regulates lifespan, and that its overexpression extends median survival and increases resistance to oxidative stress, whereas reduced expression leads to lifespan shortening. These findings underscore the biological importance of this isoform in insects and highlight the genetic and environmental dependence of SIRT6-related effects on organismal conditions [30]. The results obtained in the present study suggest that the lack of pronounced SIRT6 activity during the larval stage and early adult life (Figure 8a,b) indicates that this enzyme does not constitute a dominant component of molecular regulation at these stages, or that its function becomes apparent only in later phases of life or under different physiological conditions. This is particularly relevant given that, in *A. domesticus*, a gene homologous to mammalian SIRT6 has not yet been unequivocally described; nevertheless, the applied approach allows for a functional assessment of enzymatic activity attributed to this class of sirtuins. It should also be considered that, although *A. domesticus* represents a useful model organism, knowledge of the molecular basis of sirtuin regulation in this species remains limited, underscoring the need for further studies. The present study focused on molecular responses to RV+NDs exposure. Quantification of RV accumulation in insect tissues (e.g., using HPLC) would require dedicated analytical approaches and therefore remains an interesting direction for future studies addressing the biodistribution of RV delivered by nanodiamonds.

## 4. Materials and Methods

### 4.1. Nanodiamonds and Resveratrol

For the experiments, commercially available Single-Digit Nanodiamonds (NDs) were used in aqueous suspension form (50 mg mL^−1^) as provided by the manufacturer (PlasmaChem GmbH, Berlin, Germany), who specifies the material as detonation nanodiamonds possessing a diamond crystalline structure. As a source of resveratrol (RV), natural RV with a molecular weight of 228.24 g mol^−1^ was used (Pol-Aura^®^, Zabrze, Poland; catalogue number: PA-03-2549-P; CAS number: 501-36-0). The RV+NDs dispersion was prepared by physical adsorption. The nanodiamond suspension was first sonicated to ensure homogeneous dispersion, after which the RV solution was added and the mixture was stirred using a magnetic stirrer and subsequently sonicated for 15 min to promote interaction between RV and the ND surface.

High-resolution transmission electron microscopy (HR-TEM) was used to visualise the structural features of NDs (TITAN Cubed, Thermo Fisher Scientific, Waltham, MA, USA). HR-TEM analysis enabled visualisation of nanoscale crystalline domains and characteristic lattice fringes typical for NDs particles (Figure 12).

The comparative analysis of TEM images (TECNAI G2 X-TWIN, Thermo Fisher Scientific, USA) revealed differences in ND aggregation. Nanodiamonds alone formed looser and more porous structures (Figure 13a,a′), whereas in the presence of RV, more compact agglomerates were observed (Figure 13b,b′). Similar differences in aggregate morphology were noted at both lower (50 nm) and higher (20 nm) magnification, confirming the reproducibility of the observed effect (Figure 13a vs. Figure 13a′; Figure 13b vs. Figure 13b′).

In turn, EDS analyses revealed clear differences between NDs and RV+NDs (Figure 14). Both samples were characterised by a dominant carbon content; however, in the presence of RV, a systematic decrease in the carbon fraction was observed—from 93.68 wt% for NDs to 85.17 wt% for RV+NDs (Figure 14a,b)—accompanied by an increased contribution of elements typical of organic compounds, particularly nitrogen (2.13 wt%) (2.13 wt%) (Figure 14b). These changes, together with the TEM-observed modification in the degree of ND aggregation (Figure 1), suggest that the presence of RV may influence the system’s surface characteristics while preserving the carrier’s carbon-based nature.

**Figure 15:**
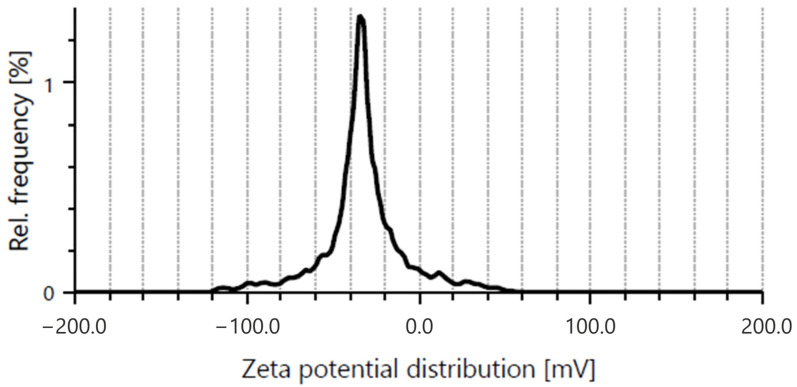
Zeta potential of RV+NDs dispersion in water (Litesizer 500, Anton Paar, Poland). Abbreviations provided in Figure 13.

This interpretation is further supported by DLS and zeta potential (Figure 15) measurements, which indicate that the addition of RV does not induce NDs aggregation, as the hydrodynamic diameter remains comparable (~126 nm). At the same time, a decrease in zeta potential from approximately −49 mV to ~−34 mV is consistent with RV interaction with the ND surface and a slight reduction in surface charge while maintaining overall system stability (Litesizer 500, Anton Paar, Warsaw, Poland) (Supplementary Figure S5).

UV–Vis spectroscopy was used to verify the absorbance characteristics of resveratrol (RV), nanodiamonds (NDs), and the RV+NDs dispersion in the wavelength range 240–900 nm. Measurements were performed using a UV–Vis spectrophotometer (TECAN Infinite M200, Tecan Austria GmbH, Grödig, Austria) with appropriate blank correction (H_2_O or H_2_O/EtOH depending on the sample composition). The obtained spectra confirmed the characteristic absorption maximum of RV at 305 nm and allowed verification that NDs do not introduce interfering absorbance in the wavelengths used in subsequent colorimetric assays (Supplementary Figure S2).

To quantify the amount of non-bound RV in supernatants after centrifugation of RV-ND mixtures, a calibration curve was constructed based on absorbance measured at λ = 305 nm using standard RV solutions. Linear regression was applied to determine the relationship between RV concentration and absorbance (Supplementary Figure S3).

Additionally, Raman spectroscopy was used to assess the structural characteristics of nanodiamonds and the RV+NDs. Raman spectra of RV, NDs, and RV+NDs were recorded using a Raman spectrometer (WITec Alpha 300 R, WITec GmbH, Ulm, Germany) equipped with a 100× objective and a 600 g mm^−1^ grating. Spectra were acquired with multiple accumulations and integration times depending on the sample (Supplementary Figure S4). The interpretation was focused on the carbon-related spectral region, with particular attention to broad features observed in the ~1340–1390 cm^−1^ and ~1580–1620 cm^−1^ ranges, which are typically used to evaluate structural disorder and surface carbon species in detonation NDs.

### 4.2. Characteristics of Acheta domesticus

*Acheta domesticus* is a valuable model organism due to its short life cycle, typically living around four months from hatching, its high reproductive rate, and its accessibility for laboratory research [90,91]. At the University of Silesia in Katowice, we have maintained a breeding programme for this species for over 30 years, as reported in our previous publications [4,92,93]. The breeding includes a long-lived strain (D), selectively bred for longevity, and a wild-type strain (H). *Acheta domesticus* is easy to maintain in laboratory conditions and exhibits well-defined developmental stages, making it suitable for studies in physiology, ageing, and developmental biology. Additionally, its relatively large size compared to other insects facilitates experimental manipulations and tissue sampling [33,91].

### 4.3. Experimental Model

All experiments were performed under standardised environmental conditions. Crickets were reared at a stable temperature of 28 ± 1 °C, with relative humidity maintained at 43.80 ± 6.72%, and subjected to a controlled light–dark cycle of 12 h each (12:12 L:D). For feeding, the insects were provided with a commercially available rabbit feed (KTD, produced by UNIPASZ, Siemiatycze, Poland), which is routinely used to sustain laboratory cultures of *Acheta domesticus* at our facility. The diet is rich in readily absorbable nutrients, with approximately 55% of its carbohydrates derived from plants. It includes proteins, fibre, fats, as well as essential minerals (such as Ca, Na, P, Zn, and Fe), and it is supplemented with vitamins, amino acids, and antioxidants. In the treatment groups, the feed was supplemented with RV at 23 mg kg^−1^ and NDs at 0.2 mg kg^−1^. The doses of RV and NDs were selected based on our previous studies conducted in the same *A. domesticus* strains under comparable experimental conditions, where both compounds were analysed separately [4,28]. Prior to feed supplementation, RV (pre-dissolved in ethanol) and NDs were co-dispersed in an aqueous solution. Before administration, all feed variants were thoroughly dried and stored in airtight containers to maintain their quality and stability. Crickets were randomly assigned to three experimental groups: Control (C), fed with the standard KTD diet; C+E, receiving feed containing ethanol (~0.01%) as a vehicle control for RV; and RV+NDs, receiving feed enriched with both RV and NDs. The experimental period spanned 22 weeks. Individuals were sampled at the larval stage and at two defined time points during adulthood: the 5th and 15th days. Intestinal tissues were dissected and processed according to previously established protocols using phosphate-buffered saline (PBS, 0.1 M, pH 7.4) [4,92]. Tissue homogenisation was performed using a Minilys homogeniser (Bertin Technologies, Montigny-le-Bretonneux, France) to obtain intestinal cell suspensions for further analyses.

### 4.4. Biochemical Analysis

#### 4.4.1. Antioxidant Enzymes Measurements

Oxidative stress markers were evaluated using a set of colorimetric assays. Lipid peroxidation levels were measured with the Lipid Peroxidation (LPO) Assay Kit (Abbexa Ltd., Cambridge, UK, abx096010), which detects lipid hydroperoxides reacting with a chromogenic substrate to indicate membrane damage. Catalase activity, responsible for neutralising hydrogen peroxide, was assessed using the Catalase Colorimetric Activity Kit (Thermo Fisher Scientific, CA, USA, EIACATC) according to the manufacturer’s protocol. The function of superoxide dismutase (SOD), crucial for converting superoxide radicals into less reactive species, was determined using the Superoxide Dismutase (SOD) Activity Assay Kit (Merck KGaA, Darmstadt, Germany, CS0009). Results were expressed as percent inhibition or enzymatic units based on a standard curve. Absorbance for all assays was measured using a TECAN Infinite M200 spectrophotometer (Tecan Austria GmbH, Grödig, Austria).

#### 4.4.2. Cell Nuclei Extraction and SIRT Activity Measurements

Nuclear proteins were extracted from *Acheta domesticus* intestinal tissue using the Nuclear Extraction Kit (Abcam, Cambridge, UK, ab113474) according to the manufacturer’s protocol. The procedure involved multiple buffer treatments and centrifugation steps, enabling the selective isolation of the nuclear fraction while minimising contamination from cytoplasmic components. Protein concentrations in the nuclear extracts were determined using the Bradford assay [94], and absorbance was measured with a UV–Vis spectrometer (TECAN Infinite M200, Tecan Austria GmbH, Grödig, Austria).

Sirtuin activity was assessed at three levels—total sirtuin activity, SIRT1, and SIRT6—each analysed using a specific assay. General sirtuin activity was measured using the Universal SIRT Activity Assay Kit (Colorimetric) (Abcam, Cambridge, UK, ab156915), which detects the deacetylation of a target substrate in the presence of NAD^+^ through a colorimetric change, as recorded via spectrophotometry. For isoform-specific analyses, the SIRT1 Activity Assay Kit (fluorometric) (Abcam, Cambridge, UK, ab156065) and the SIRT6 Activity Assay Kit (fluorometric) (Abcam, Cambridge, UK, ab156068) were employed. These assays use fluorescent substrates that, upon enzymatic deacetylation, release a fluorophore. Fluorescence intensity was measured using a Fluorescence Spectrometer Plate Reader (HITACHI F-7000, Hitachi, Ltd., Tokyo, Japan), allowing for sensitive quantification of enzyme activity.

#### 4.4.3. DNA Damage Measurements

DNA damage was assessed using the Muse^®^ Multi-Colour DNA Damage Kit (Luminex Corporation, Austin, TX, USA, Part Number: MCH200107), which allows concurrent evaluation of ATM and H2A.X activation. The assay is based on phospho-specific, directly conjugated antibodies targeting ATM (Ser1981)-PE and Histone H2A.X PECy5, enabling detection of their phosphorylation status. Sample preparation and staining were performed according to the manufacturer’s protocol, including fixation, permeabilisation, and antibody incubation. Following washing and resuspension in assay buffer, measurements were obtained using the Guava^®^ Muse^®^ cell analyser (Luminex Corporation, TX, USA).

### 4.5. Data Visualisation and Statistical Procedures

Kaplan–Meier survival analysis was used to evaluate survival in the experimental groups, with survival probabilities over time presented as survival curves. The variance of the Kaplan–Meier survival estimator was calculated using Greenwood’s formula. Differences between groups were assessed using the Log-Rank test (Chi-squared, *p* < 0.05). The analysis was conducted on the wild-type strain (H) and the long-lived strain (D) over 147 days. Individuals were maintained separately within each experimental group, with approximately *n* ~ 80 individuals per strain (Python, lifelines package).

Data visualisation and statistical analyses were performed using Python (version 3.13.2). Data processing was conducted with the following Python libraries: Pandas (version 2.2.3) for data management, NumPy (version 2.2.4) for numerical computations, and Matplotlib.pyplot (version 3.10.1) for graphical representation. Experimental datasets were imported from CSV files, and group labels and measurement timepoints were standardised to ensure clarity and consistency in subsequent analyses. Descriptive statistics, including means and standard errors of the mean (SEM), were calculated for each strain, treatment group, and time point. The results were visualised as grouped bar plots, enabling direct comparison across experimental conditions.

Preliminary statistical assumptions were verified using STATISTICA^®^ 13 (TIBCO Software Inc., Palo Alto, CA, USA), where the data were tested for normality (*p* < 0.05) and homogeneity of variances using Levene’s test. LPO, CAT, SOD, percentage inhibition, total sirtuin activity, SIRT1, SIRT6, pATM, γH2A.X, and DSBs were quantified using five independent biological replicates per treatment group (*n* = 5 per group: C, C+E, RV+NDs). Subsequent multivariate analyses were conducted in Python (version 3.13.2). Group-level differences were assessed using PERMANOVA (Permutational Multivariate Analysis of Variance) with 999 permutations, followed by PERMDISP to assess multivariate dispersion homogeneity. PERMANOVA revealed significant effects (*p* < 0.05), whereas PERMDISP was not significant (*p* ≥ 0.05), confirming that observed differences were not driven by variance heterogeneity. Associations between sirtuin activity and DNA damage markers were assessed using Spearman’s rank correlation (r_s_; *p* < 0.05), calculated separately for strains H and D (SciPy, Python 3.13.2). These analyses were conducted using the Pandas, NumPy, SciPy, scikit-bio, and tqdm libraries. All pairwise comparisons were automated, and the resulting matrices were exported to Excel (Microsoft, Redmond, WA, USA) for further interpretation. The complete analytical workflow was implemented and executed in the Jupyter Notebook (version 4.3.6) environment (Project Jupyter, San Luis Obispo, CA, USA).

## 5. Conclusions

The overall results indicate that hypothesis H1 was only partially confirmed, and this effect was strictly dependent on the genetic background of the studied organism. The simultaneous administration of resveratrol and nanodiamonds (RV+NDs) led to a sustained increase in total sirtuin activity exclusively in the long-lived strain of *Acheta domesticus* (strain D), without clear selective activation of SIRT1 or SIRT6 and without a universal translation into lifespan extension. These findings indicate that modulation of sirtuin activity by RV+NDs is context-dependent and does not constitute a simple pro-longevity mechanism.

Hypothesis H2 was not unequivocally confirmed, as combined RV+NDs administration did not result in a persistent enhancement of oxidative stress. The observed changes in parameters were transient and age- and strain-dependent, indicating the absence of a cumulative pro-oxidative effect of this combination. Further studies are therefore required, particularly within a single species.

At the same time, it was demonstrated that the most sensitive indicators of the response to combined RV+NDs administration were markers of the DNA damage response and total sirtuin activity, whereas classical oxidative stress parameters as well as SIRT1 and SIRT6 activity exhibited greater stability and a stronger dependence on organismal age. This suggests that the RV+NDs combination induces a qualitatively distinct molecular response profile, involving activation of adaptive mechanisms rather than progressive cellular damage.

These findings also highlight the importance of considering genetic background and organismal age when evaluating nanodelivery strategies aimed at modulating ageing-related pathways. The results suggest that nanodiamond-based systems may alter molecular responses to bioactive compounds without necessarily producing universal longevity effects. Future studies should therefore address long-term exposure, biodistribution, and tissue accumulation of nanodiamonds, as well as the bioavailability and release kinetics of resveratrol delivered by nanocarriers.

In an era of growing interest in life-extending strategies and the search for factors modulating the rate of ageing, the present study highlights the need for cautious interpretation of the effects of long-term supplementation with substances considered potentially pro-longevity. Moreover, it indicates that even compounds with a presumed beneficial and safe action profile may, when used chronically, induce complex and context-dependent molecular responses whose biological consequences are not always unequivocally favourable. These studies constitute a valuable contribution to knowledge on the biological effects of combined application of bioactive compounds and nanocarriers in the insect model organism *Acheta domesticus*, for which the mechanisms regulating sirtuins and molecular stress responses remain poorly understood. Such an approach may lead to subtle yet biologically significant modulation of molecular processes without disrupting cellular homeostasis.

## Data Availability

Raw data are provided on the RepOD database; DOI: https://doi.org/10.18150/CFSPUX.

## Supplementary Materials

The following supporting information can be downloaded at https://www.mdpi.com/article/10.3390/ijms27062786/s1.

## Abbreviations

The following abbreviations are used in this manuscript:

AMPK	AMP-activated protein kinase
ATM	Ataxia telangiectasia mutated kinase
AUC	Area under the curve
CAT	Catalase
DDR	DNA damage response
DSBs	Double-strand breaks
EDS	Energy-dispersive X-ray spectroscopy
H	Wild-type strain
D	Longevity-selected strain
H2A.X (γH2A.X)	Phosphorylated histone H2A.X
L:D	Light–dark cycle
LPO	Lipid peroxidation
NAD^+^	Nicotinamide adenine dinucleotide (oxidised form)
NDs	Nanodiamonds
PBS	Phosphate-buffered saline
PERMANOVA	Permutational multivariate analysis of variance
PERMDISP	Permutational analysis of multivariate dispersions
pATM	Phosphorylated ATM
ROS	Reactive oxygen species
RV	Resveratrol
SEM	Scanning electron microscopy
SEM (statistics)	Standard error of the mean
SIRT	Sirtuin
SIRT1	Sirtuin 1
SIRT6	Sirtuin 6
SOD	Superoxide dismutase
TEM	Transmission electron microscopy
wt%	Weight percent
