# Supplementary material for: Evaluation of Molecular Responses and Longevity Markers in Acheta domesticus Following Combined Resveratrol and Nanodiamond Exposure
Source: Int J Mol Sci. 2026 Mar 19;27(6):2786. doi: 10.3390/ijms27062786 (PMC13026565; doi:10.3390/ijms27062786)
Supplement: Supplementary file 1 [file ijms-27-02786-s001.zip › Supplementary_materials.pdf]

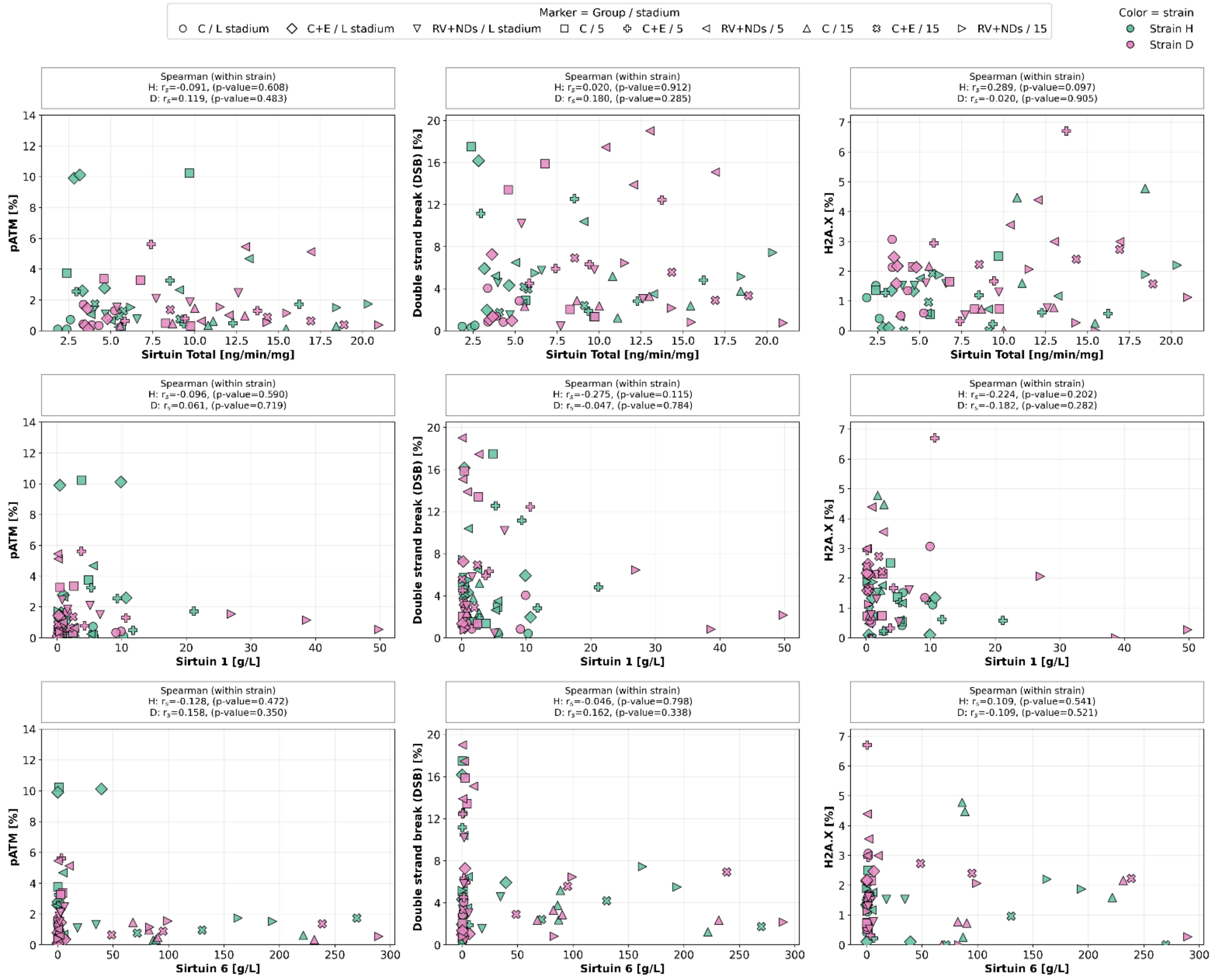

Figure S1. Spearman correlation analysis between sirtuin activity (Sirtuin total, Sirtuin 1, and Sirtuin 6) and DNA damage response markers (pATM, H2A.X, and DSB) in *Acheta domesticus*. Correlation coefficients (r_s) and corresponding p-values were calculated separately for the wild-type strain (H) and the long-lived strain (D). Each point represents an individual measurement from experimental groups (C, C+E, RV+NDs) across different developmental stages (L stadium, Day 5, Day 15). Statistical analyses and visualisation were performed in Python (version 3.13.2) using the Pandas, NumPy, SciPy, and Matplotlib libraries.

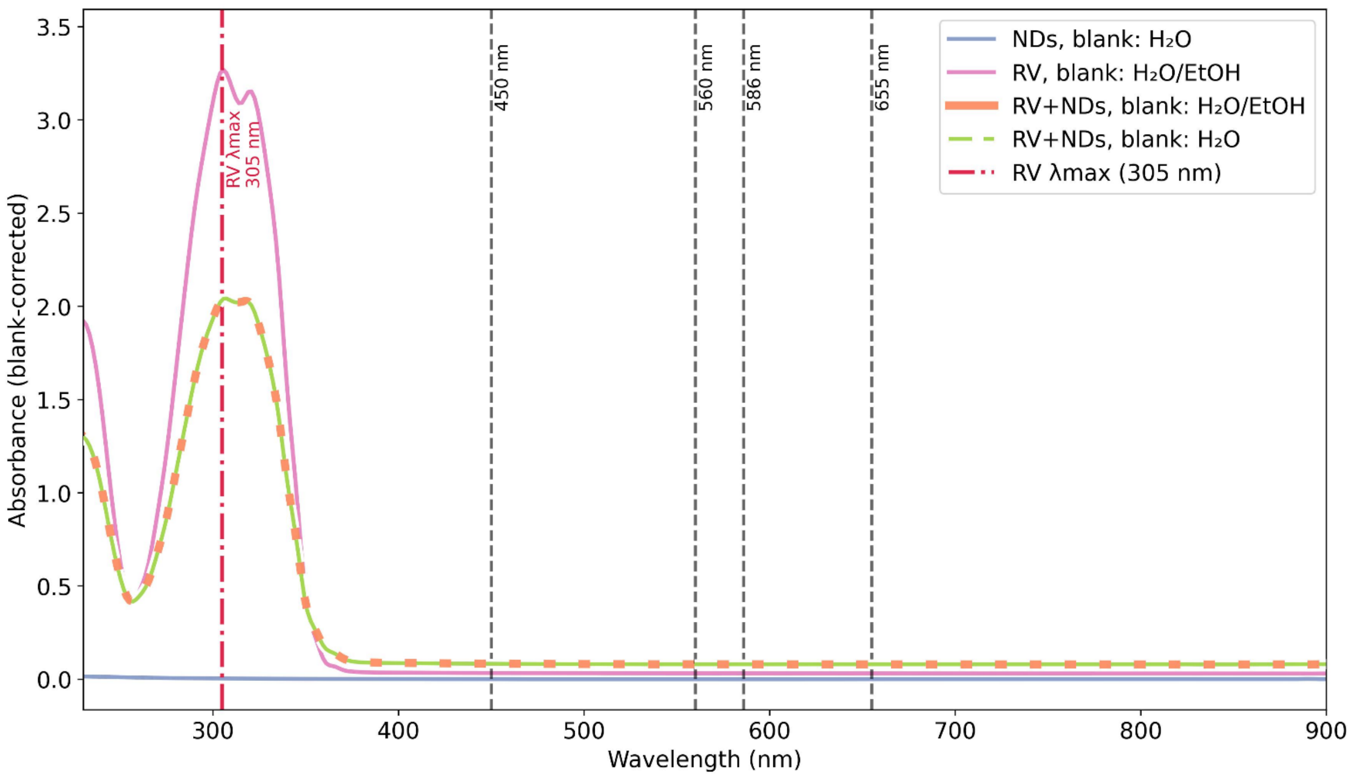

Figure S2. UV-Vis absorption spectra of nanodiamonds (NDs), resveratrol (RV), and the RV-NDs mixture after blank correction. Measurements were performed using a UV-Vis spectrometer (TECAN Infinite M200, Tecan Austria GmbH, Grödig, Austria) over the spectral range of 240-900 nm, with a 5 nm scanning interval. Spectra are shown for NDs (0.2 mg kg^{-1} feed) measured against a water blank (H_2O), RV (23 mg kg^{-1} feed) measured against a water-ethanol blank (H_2O/EtOH), and the RV+NDs mixture (23 mg kg^{-1} RV + 0.2 mg kg^{-1} NDs) corrected either with an H_2O/EtOH blank or with an H_2O blank. The dashed grey vertical lines indicate wavelengths used for spectrophotometric assays (450, 560, 586, and 655 nm). The red dashed line marks the maximum absorption wavelength of RV (λmax = 305 nm). Absorbance values represent mean spectra after blank subtraction. The plot was generated in Python (version 3.13.2; NumPy, Pandas, Matplotlib, SciPy).

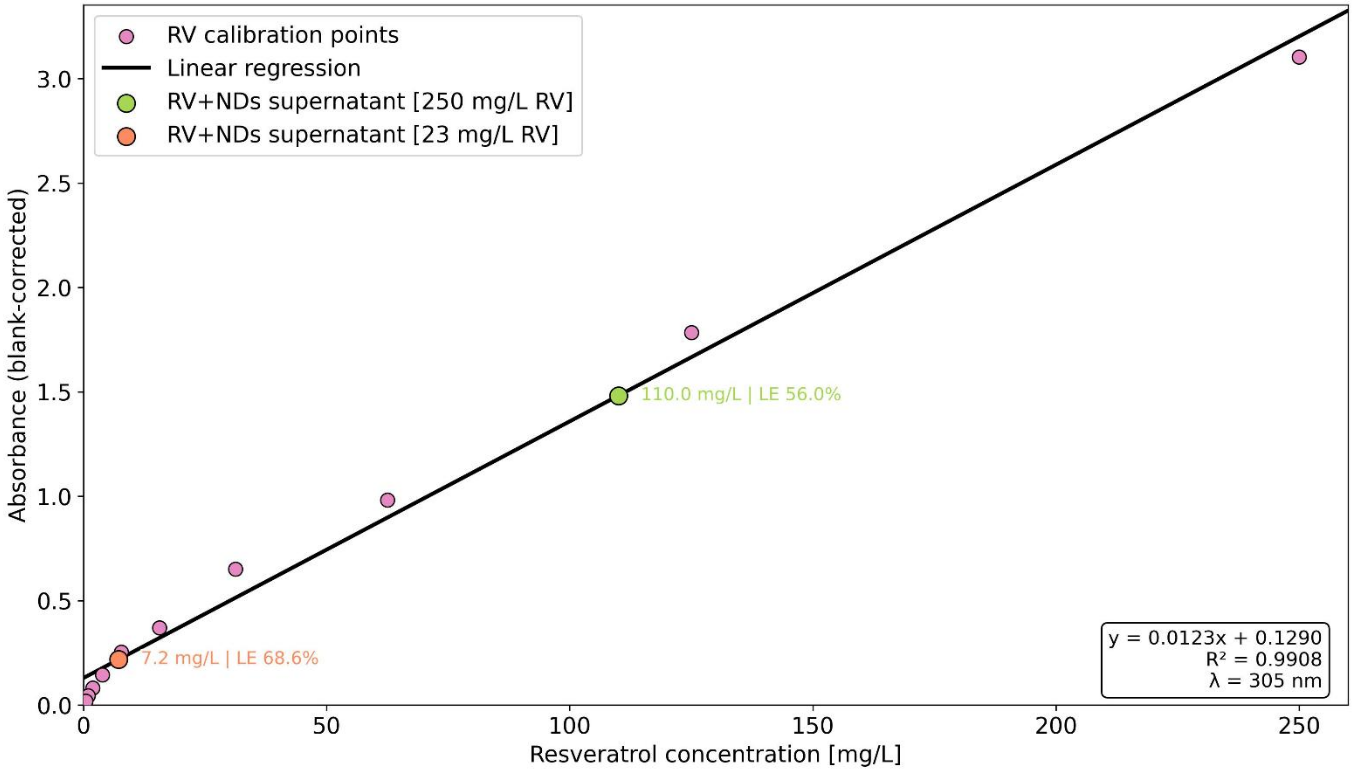

Figure S3. Calibration curve of resveratrol (RV) determined from UV-Vis absorbance at λ = 305 nm after blank correction. Standard solutions of RV were prepared in the concentration range of 0.49-250 mg L^{-1} and measured using a UV-Vis spectrometer (TECAN Infinite M200, Tecan Austria GmbH, Grödig, Austria). The linear regression equation obtained for the calibration curve was y = 0.0123x + 0.1290 (R^2 = 0.9908). The calculated concentrations of non-bound RV in the supernatants obtained after centrifugation of RV-NDs mixtures are indicated on the plot 250 mg L^{-1} RV with 2 mg kg^{-1} nanodiamonds (NDs) and 23 mg L^{-1} RV with 0.2 mg kg^{-1} NDs, respectively. Loading efficiency (LE) of RV on nanodiamonds (NDs) was determined based on the difference between the initial RV concentration and the concentration of free RV in the supernatant. The plot was generated in Python (version 3.13.2; NumPy, Matplotlib, SciPy).

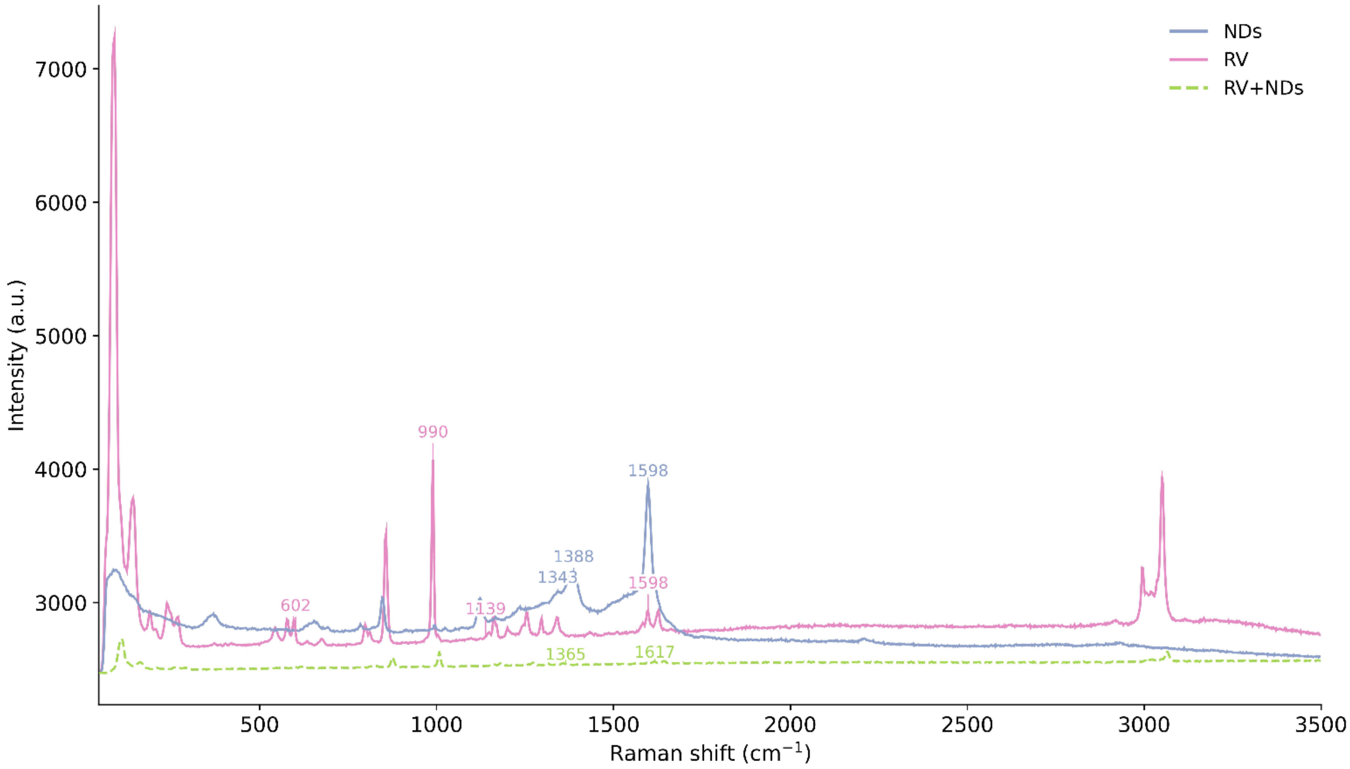

Figure S4. Raman spectra of resveratrol (RV), nanodiamonds (NDs), and the RV+NDs recorded using a Raman spectrometer (WITec Alpha 300 R, WITec GmbH, Ulm, Germany). Measurements were performed using a 100× objective (Zeiss LD EC Epiplan-Neofluar, NA 0.75) and a 600 g mm^{-1} grating. The excitation wavelength was 531.951 nm for RV and NDs, and 487.982 nm for the RV+NDs sample. Spectra were collected with 10 accumulations and integration times of 5 s for RV and 3 s for NDs and RV+NDs. The Rayleigh scattering region below 45 cm^{-1} was excluded from the spectra prior to visualization. The spectra were processed and plotted in Python (version 3.13.2; NumPy, Matplotlib, SciPy).

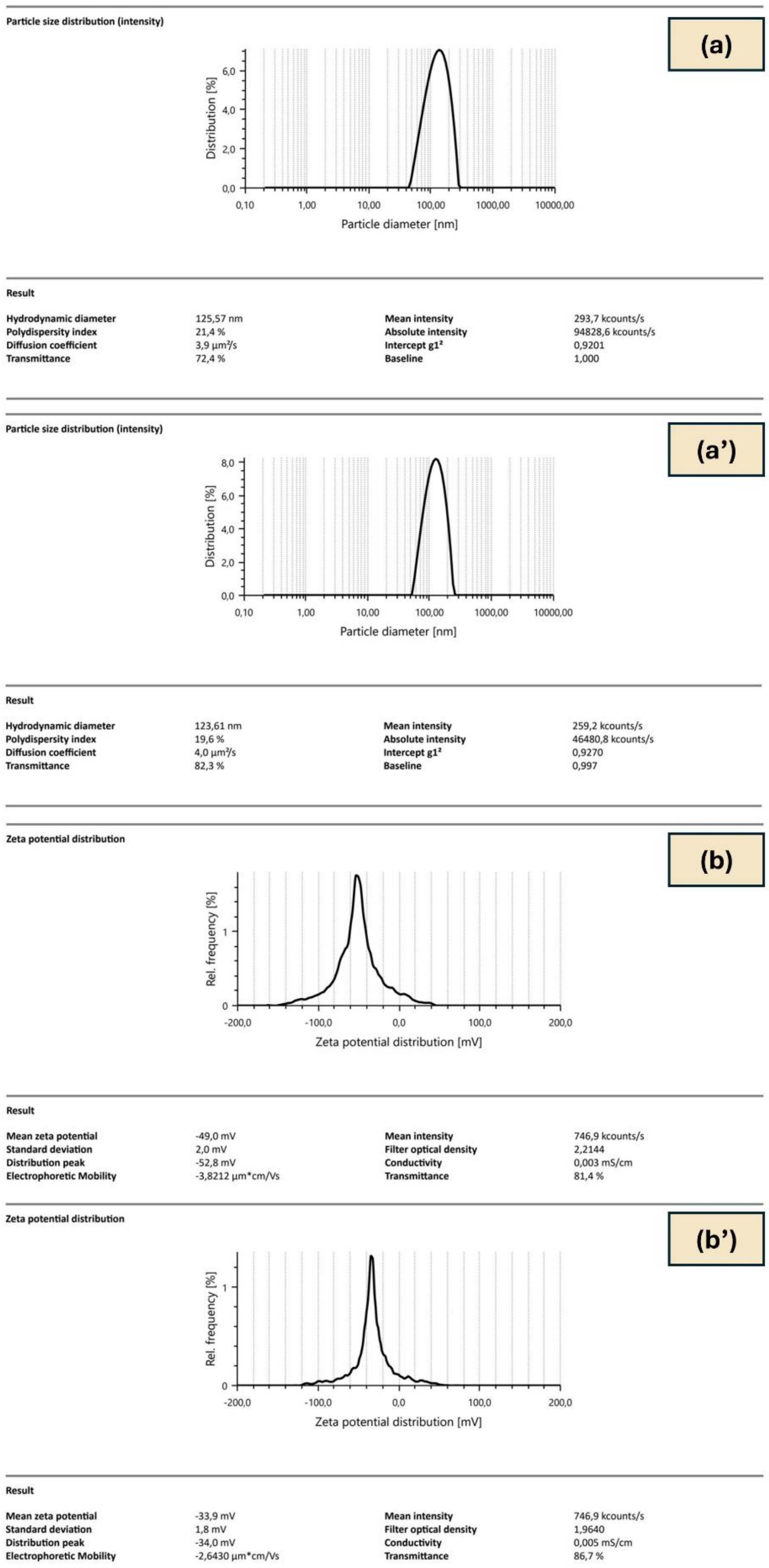

Figure S5. Particle size distribution and zeta potential of nanodiamonds (NDs) and the RV+NDs system measured by dynamic light scattering (DLS). (a) Hydrodynamic diameter distribution of NDs and (a′) RV+NDs dispersion. (b) Zeta potential distribution of NDs and (b′) RV+NDs (Litesizer 500, Anton Paar, Poland).
